# Supplementary material for: Synaptic metaplasticity in binarized neural networks
Source: Nat Commun. 2021 May 5;12:2549. doi: 10.1038/s41467-021-22768-y (PMC8100137; doi:10.1038/s41467-021-22768-y)
Supplement: Supplementary file 1 — Supplementary Information [file 41467_2021_22768_MOESM1_ESM.pdf]

Supplementary Information : Synaptic Metaplasticity in Binarized Neural Networks

Axel Laborieux[1,*], Maxence Ernoult[1,2], Tifenn Hirtzlin[1], and Damien Querlioz[1,*]

[1]Université Paris-Saclay, CNRS, Centre de Nanosciences et de Nanotechnologies, 91120, Palaiseau, France.
[2]Unité Mixte de Physique, CNRS, Thales, Université Paris-Saclay, 91120, Palaiseau, France.
[*]axel.laborieux@c2n.upsaclay.fr, damien.querlioz@c2n.upsaclay.fr

Supplementary Note 1: Forward and Backward Propagation in Binarized Neural Networks

Supplementary Algorithm 1 Forward function of the BNN reproduced from [1]. $\mathbf{W^b} = (\mathbf{W^b}_l)_{l=1...L}$ are the binary weights, $\boldsymbol{\theta}^{\mathrm{BN}} = \{(\boldsymbol{\gamma}_l, \boldsymbol{\beta}_l) \mid l = 1...L\}$ are Batch Normalization parameters. L is the total number of layers and the subscript l when specified is the layer index. $\mathbf{x}$ is a batch of input data with dimensions (P,N) with P the number of pixels and N the number of examples in the batch. $\mathrm{E}(\cdot)$ and $\mathrm{Var}(\cdot)$ are batch-wise mean and variance. While they are computed during training with the statistics of the batches, running averages of the mean and variance are stored to be used at test time. This enables the network to infer on a single example at test time. ε is a small number to avoid division by zero, it was set to 10^{-5} in all the experiments.

Input: $\mathbf{W^b}$, $\boldsymbol{\theta}^{\mathrm{BN}}$, $\mathbf{x}$.
Output: $\hat{\mathbf{y}}$, cache.

$\mathbf{a}_0 \leftarrow \mathbf{x}$ ▷ Input is not binarized
for $l = 1$ to L **do** ▷ For loop over the layers
  $\mathbf{z}_l \leftarrow \mathbf{W^b}_l \mathbf{a}_l$ ▷ Matrix multiplication
  $\mathbf{a}_l \leftarrow \boldsymbol{\gamma}_l \cdot \frac{\mathbf{z}_l - \mathrm{E}(\mathbf{z}_l)}{\sqrt{\mathrm{Var}(\mathbf{z}_l)+\varepsilon}} + \boldsymbol{\beta}_l$ ▷ Batch Normalization [2]
  if $l < L$ **then** ▷ If not the last layer
    $\mathbf{a}^{\mathrm{b}}_l \leftarrow \mathrm{Sign}(\mathbf{a}_l)$ ▷ Activation is binarized
  end if
end for
$\hat{\mathbf{y}} \leftarrow \mathbf{a}_L$
return $\hat{\mathbf{y}}$, cache

Supplementary Algorithm 2 Backward function of the BNN reproduced from [1]. $\mathbf{W^b} = (\mathbf{W^b}_l)_{l=1...L}$ are the binary weights, $\boldsymbol{\theta}^{\mathrm{BN}} = \{(\boldsymbol{\gamma}_l,\ \boldsymbol{\beta}_l) \mid l = 1...L\}$ are Batch Normalization parameters. BackBatchNorm$(\cdot)$ specifies how to backpropagate through the Batch normalization [2]. L is the total number of layers and the subscript l when specified is the layer index. $1_{|a_l|\leq 1}$ is the derivative of Hardtanh taken as a replacement for back propagating through Sign activation.

Input: $C, \hat{\mathbf{y}}, \mathbf{W^b}, \boldsymbol{\theta}^{\mathrm{BN}}$, cache.
Output: $(\partial_W C, \partial_\theta C)$.

$\mathbf{g}_{a_L} \leftarrow \frac{\partial C}{\partial \hat{\mathbf{y}}}$ ▷ Cost gradient with respect to output
for $l = L$ to 1 **do** ▷ For loop backward over the layers
  if $l < L$ **then** ▷ If not the last layer
    $\mathbf{g}_{a_l} \leftarrow \mathbf{g}_{a_l^{\mathrm{b}}} \cdot 1_{|a_l|\leq 1}$ ▷ Back Prop through Sign
  end if
  $(\mathbf{g}_{z_l}, \mathbf{g}_{\gamma_l}, \mathbf{g}_{\beta_l}) \leftarrow \mathrm{BackBatchNorm}(\mathbf{g}_{a_l}, \mathbf{z}_l, \boldsymbol{\gamma}_l, \boldsymbol{\beta}_l)$ ▷ See [2]
  $\mathbf{g}_{a_{l-1}^{\mathrm{b}}} \leftarrow \mathbf{W^b}_l \mathbf{g}_{z_l}$
  $\mathbf{g}_{W_l^{\mathrm{b}}} \leftarrow {\mathbf{a}_{l-1}^{\mathrm{b}}}^\top \mathbf{g}_{z_l}$
end for
$\partial_W C \leftarrow \{\mathbf{g}_{W_l^{\mathrm{b}}} \mid l = 1...L\}$
$\partial_\theta C \leftarrow \{\mathbf{g}_{\gamma_l},\ \mathbf{g}_{\beta_l} \mid l = 1...L\}$
return $(\partial_W C, \partial_\theta C)$

The optimization is performed using Adaptive Moment Estimation (Adam) algorithm [3]. As the sign function is not differentiable in zero and the derivative is zero on $\mathbb{R}^*$, during error backpropagation the derivative of hardtanh function is used as a replacement for the derivative of the Sign function. The activation function is the sign function except for the output layer. The input neurons are not binarized. We use batch normalization [2] at all layers as detailed in Alg. 1. The following derivation for layer l,

$$\gamma_l \cdot \frac{z - \mathrm{E}(z)}{\sqrt{\mathrm{Var}(z)+\varepsilon}} + \beta_l = \frac{\gamma_l}{\sqrt{\mathrm{Var}(z)+\varepsilon}} \left(z - \left[\mathrm{E}(z) - \frac{\beta_l \sqrt{\mathrm{Var}(z)+\varepsilon}}{\gamma_l} \right] \right)$$

$$a = \mathrm{Sign}(\gamma_l)\mathrm{Sign}\left(z - \left[\mathrm{E}(z) - \frac{\beta_l \sqrt{\mathrm{Var}(z)+\varepsilon}}{\gamma_l} \right] \right)$$

shows that because the Sign function is invariant by any multiplicative constant in the input, the only task dependent parameters we need to store for an inference hardware chip is the term between square brackets, along with the sign of γ_l. The amount of task dependent parameters scales as the number of neurons and is order of magnitudes smaller than the number of synapses.

Adam optimizer updates the hidden weight with loss gradients computed using binary weights only. We use a small weight decay of 10^{-7} in the Adam optimizer to make zero floating values more stable. However, consolidated weights are not subject to weight decay, as we implement weight decay as a modification of the loss gradient, which is gradually suppressed by f_{meta}.

Supplementary Note 2: Training parameters

The batch normalization layers parameters were not learned for the Fashion MNIST experiment whereas they were learned for the CIFAR-10 experiment.

The batch normalization parameters are set to $\beta = 0$, $\gamma = 1$ for the Fashion MNIST experiment. The performance of the BNN with learned batch normalization parameters was inferior, as batch normalization parameters appear to overfit to the subsets of data. In the CIFAR-10 experiment the performance was higher with learned batch normalization parameters. The architecture of VGG-7 network consists of 6 convolutional layers of 3×3 sized

	pMNISTs		
Network	Binarized meta	Binarized EWC	Full precision
Layers	784-4096-4096-10	784-4096-4096-10	784-4096-4096-10
Learning rate	0.005	0.005	0.005
Minibatch size	100	100	100
Epochs/task	40	40	40
m	1.35	0.0	1.35
λ_{EWC}	0.0	5,000	0.0
Weight decay	1e-7	1e-7	1e-7
Initialization	Uniform width = 0.1	Uniform width = 0.1	Uniform width = 0.1

Supplementary Table 1. Hyperparameters for the permuted MNISTs experiment.

	FMNIST - MNIST
Network	Binarized meta
Layers	784-4096-4096-10
Learning rate	0.005
Minibatch size	100
Epochs/task	50
m	1.5
Weight decay	1e-8
Initialization	Uniform width = 0.1

Supplementary Table 2. Hyperparameters for the permuted FMNIST-MNIST experiment.

	Stream FMNIST	Stream CIFAR-10
Network	Binarized meta	Binarized meta
Layers	784-1024-1024-10	VGG-7
Sub Parts	60	20
Learning rate	0.005	0.0001
Minibatch size	100	64
Epochs/subset	20	200
m	2.5	13.0
Weight decay	1e-7	0.0
Initialization	Uniform width = 0.1	Gauss width = 0.007

Supplementary Table 3. Hyperparameters for the stream learning experiment.

kernels with kernel number per layer following the sequence 128-128-256-256-512-512. The classifier consists of two hidden layers of 2048-1024 hidden units. Dropout was used in the classifier with value 0.5.

Supplementary Note 3: Implementation of Synaptic Intelligence

In this Supplementary Note, we discuss the implementation of the synaptic intelligence algorithm [4], designed for continual learning in full precision neural networks. The algorithm consists in optimizing the loss function

$$\widetilde{L}_\mu = L_\mu + c\sum_k \Omega_k^\mu (\widetilde{\theta}_k - \theta_k)^2 \tag{1}$$

when learning the task μ, where L_μ is the loss function associated with the current task and $c\sum_k \Omega_k^\mu (\widetilde{\theta}_k - \theta_k)^2$ is a "surrogate loss"[4] compelling the current parameters θ_k to stay close to the parameters $\widetilde{\theta}_k$ optimized for previous tasks. Ω_k^μ is the importance factor for parameter $\widetilde{\theta}_k$ and is updated between each task by

$$\Omega_k^\mu = \sum_{\nu<\mu} \frac{\omega_k^\nu}{(\Delta_k^\nu)^2 + \xi}. \tag{2}$$

Δ_k^ν is a normalization factor equal to the total parameter change over the latest learned task, and ξ is a small constant number avoiding any division by zero. ω_k^ν is computed in an online fashion by approximating the path integral of the parameters and can be interpreted as the parameter specific contribution to changes in the total loss.

$$\omega_k^\mu = -\sum_t \frac{\partial L}{\partial \theta_k(t)} (\theta_k(t+1) - \theta_k(t)). \tag{3}$$

As a control experiment, we reproduce the results of [4] for the permuted MNIST benchmark in Suppl. Fig. 2a, with $c = 0.1$ and $\xi = 0.1$. In the case of binarized neural networks, we tried several ways of computing the importance factor Ω_k^μ by employing either the binarized weight or the hidden weight for ω_k^μ and Δ_k^ν. The best performance was achieved by using the binarized weight values for ω_k^μ and the hidden weight values for Δ_k^ν and $c = 1.0$, $\xi = 0.1$. The results are shown in Supp. Fig. 2b.

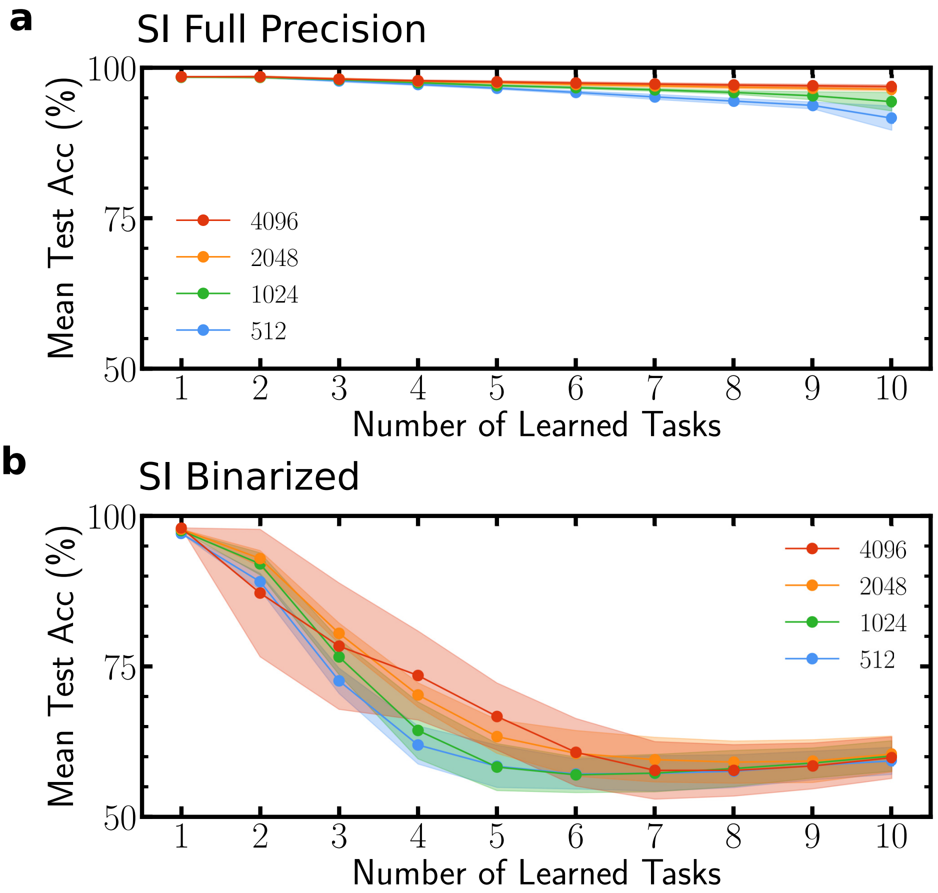

Supplementary Figure 1. Synaptic Intelligence a applied to full precision neural networks with two hidden ReLU layers of increasing size ranging from 512 to 4,096 for the permuted MNIST benchmark, results reproduced from [4]. **b** The best performing adaptation of Synaptic Intelligence to binarized neural networks (see Suppl. Note .) The curves are averaged over five runs and shadows stand for one standard deviation.

Supplementary Note 4: Use of a Metaplasticity Function f_{meta} Featuring a Hard Threshold

In this note, we present a control experiment where the modulating function f_{meta} is a hard threshold function such that $f_{\text{meta}}(W^{\text{h}}) = 1$ if $|W^{\text{h}}| < m$, and $f_{\text{meta}}(W^{\text{h}}) = 0$ if $|W^{\text{h}}| > m$. The hyperparameter m is, in this case, the threshold value above which f_{meta} is zero. The value of m is obtained by hyperparameter tuning and set to $m = 0.4$.

We observe that the performance is degraded modestly when using such threshold mechanism, in accordance with the theoretical evidence that high hidden weights correspond to important binarized weights for consolidation. The most degradation is observed in the regime where the neural network exhibits the highest capacity in number of tasks (network with 4,096-wide layers trained with nine or ten tasks).

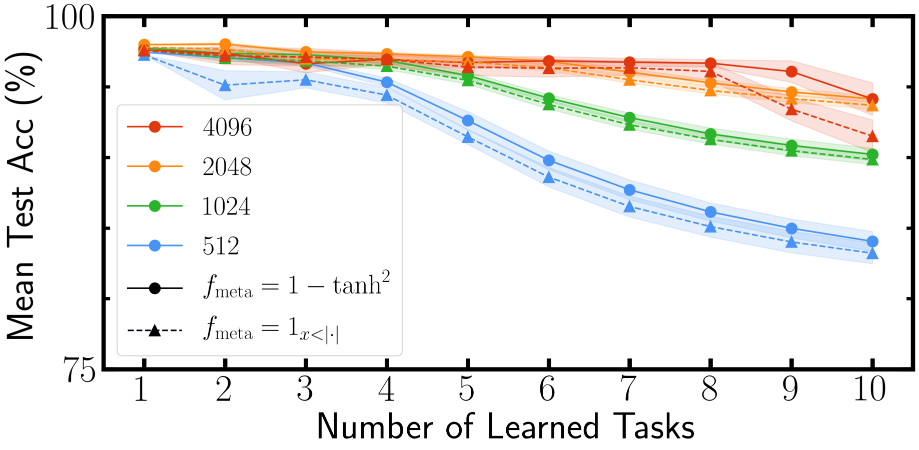

Supplementary Figure 2. Comparison of different choices for f_{meta}, when ten training ten permuted MNIST tasks. This plot shows the comparison between two classes of f_{meta} functions. The bullets represent the metaplastic BNNs with the function class introduced in the body text with $m = 1.35$, while the squares denote an f_{meta} function with a hard threshold above which a weight is irreversibly consolidated. The threshold value is tuned to be 0.4. The colors denote increasing network sizes. The curves are averaged over five runs and shadows stand for one standard deviation.

Supplementary Note 5: Mathematical proofs

Definition 1 (Quadratic Binary Task)*:* Consider the loss function:

$$\mathscr{L}(\mathbf{W}) = \frac{1}{2}(\mathbf{W}-\mathbf{W}^*)^T \cdot \mathbf{H} \cdot (\mathbf{W}-\mathbf{W}^*) \tag{4}$$

with a symmetric definite positive matrix $\mathbf{H} \in \mathbb{R}^{d\times d}$. Gradients are given by $\mathbf{g}(\mathbf{W}) = \mathbf{H}\cdot(\mathbf{W}-\mathbf{W}^*)$. We assume the following optimization scheme:

$$\mathbf{W^h}_{t+1} = \mathbf{W^h}_t - \eta \mathbf{H}\cdot(\mathrm{sign}(\mathbf{W^h}_t) - \mathbf{W}^*), \tag{5}$$

where sign returns the sign of a vector component-wise.

Lemma 1 (Condition for hidden Weight confinement)*:* Let $\mathbf{W^h}$ optimize a quadratic binary task according to the dynamics $\mathbf{W^h}_{t+1} = \mathbf{W^h}_t - \eta\mathbf{H}(\mathrm{sign}(\mathbf{W^h}_t) - \mathbf{W}^*)$. Let $\mathscr{B}_\infty$ be the unit ball for the infinite norm and $\overline{\mathscr{B}_\infty}$ its closure. Then:

$$\mathbf{W}^* \in \mathscr{B}_\infty \Rightarrow \exists C>0, \forall t \in \mathbb{N}, \|\mathbf{W^h}_t\|_\infty < C \tag{6}$$

$$\mathbf{W}^* \notin \overline{\mathscr{B}_\infty} \Rightarrow \lim_{t\to\infty} \|\mathbf{W^h}_t\|_\infty = \infty \tag{7}$$

Proof of Lemma 1. We first prove Eq. (7). Let us assume that $\mathbf{W}^* \notin \overline{\mathscr{B}_\infty}$ so that there exists at least one component $i \in [\![1,d]\!]$ such that $|W_i^*| > 1$. Since H is symmetric definite positive, it is invertible. Taking the euclidian scalar product between $\mathbf{H}^{-1}\mathbf{e}_i$ and the update $(\mathbf{W^h}_{t+1} - \mathbf{W^h}_t)$ yields:

$$\begin{aligned}
\langle \mathbf{H}^{-1}\mathbf{e}_i,\ \mathbf{W^h}_{t+1} - \mathbf{W^h}_t\rangle &= (\mathbf{H}^{-1}\mathbf{e}_i)^T \cdot (\mathbf{W^h}_{t+1} - \mathbf{W^h}_t) \\
&= -\eta(\mathbf{H}^{-1}\mathbf{e}_i)^T \cdot \mathbf{H}(\mathrm{sign}(\mathbf{W^h}_t) - \mathbf{W}^*) \\
&= -\eta\ \mathbf{e}_i^T \cdot (\mathbf{H}^{-1})^T\mathbf{H}(\mathrm{sign}(\mathbf{W^h}_t) - \mathbf{W}^*) \\
&= -\eta\ \mathbf{e}_i^T \cdot \mathbf{H}^{-1}\mathbf{H}(\mathrm{sign}(\mathbf{W^h}_t) - \mathbf{W}^*) \\
&= -\eta\ \mathbf{e}_i^T \cdot (\mathrm{sign}(\mathbf{W^h}_t) - \mathbf{W}^*) \\
&= -\eta(\mathrm{sign}(W^{\mathrm{h}}_{i,t}) - W_i^*),
\end{aligned}$$

where we have used at the fourth equality that $\mathbf{H}^{-1}$ is also symmetric. Since $|W_i^*| > 1$, the sign of $\mathrm{sign}(W^{\mathrm{h}}_{i,t}) - W_i^*$ is constant (and $\neq 0$), so the component of $\mathbf{W}$ along $\mathbf{H}^{-1}\mathbf{e}_i$ is expected to diverge. More precisely, let us assume $W_i^* > 1$ so that $\mathrm{sign}(W^{\mathrm{h}}_{i,t}) - W_i^* < 1 - W_i^*$ and:

$$\langle \mathbf{H}^{-1}\mathbf{e}_i, \mathbf{W^h}_{t+1} - \mathbf{W}^{\mathrm{h}}_t\rangle \geq -\eta(1 - W_i^*). \tag{8}$$

Summing Eq. (8) from time step 0 to t yields:

$$\langle \mathbf{H}^{-1}\mathbf{e}_i, \mathbf{W^h}_t\rangle \geq -\eta(1 - W_i^*)t + \langle \mathbf{H}^{-1}\mathbf{e}_i, \mathbf{W^h}_0\rangle, \tag{9}$$

showing that $\lim_{t\to+\infty}\langle \mathbf{H}^{-1}\mathbf{e}_i, \mathbf{W^h}_t\rangle = +\infty$. Consequently there exists $j \in [\![1,d]\!]$ such that $\lim_{t\to+\infty}\langle \mathbf{e}_j, \mathbf{W^h}_t\rangle = +\infty$ and therefore $\lim_{t\to\infty}\|\mathbf{W^h}_t\|_\infty = +\infty$. Similarly if $W_i^* < -1$, we show that:

$$\langle \mathbf{H}^{-1}\mathbf{e}_i, \mathbf{W^h}_t\rangle \leq \eta(1 + W_i^*)t + \langle \mathbf{H}^{-1}\mathbf{e}_i, \mathbf{W^h}_0\rangle, \tag{10}$$

giving the same conclusion as above.

We now prove Eq. (6). Let us assume that $\mathbf{W}^* \in \mathscr{B}_\infty$, i.e. $\forall i \in [\![1,d]\!]$, $|W_i^*| < 1$. We have:

$$
\begin{aligned}
&\|\mathbf{W^h}_{t+1}\|^2_{\mathbf{H}^{-1}} = \langle \mathbf{W^h}_{t+1}, \mathbf{W^h}_{t+1}\rangle_{\mathbf{H}^{-1}}\\
&= \langle \mathbf{W^h}_t + \Delta\mathbf{W^h}_t, \mathbf{W^h}_t + \Delta\mathbf{W^h}_t\rangle_{\mathbf{H}^{-1}}\\
&= \|\mathbf{W^h}_t\|^2_{\mathbf{H}^{-1}} + 2\langle \Delta\mathbf{W^h}_t, \mathbf{W^h}_t\rangle_{\mathbf{H}^{-1}} + \langle \Delta\mathbf{W^h}_t, \Delta\mathbf{W^h}_t\rangle_{\mathbf{H}^{-1}}\\
&= \|\mathbf{W^h}_t\|^2_{\mathbf{H}^{-1}} + 2\langle \mathbf{H}^{-1}\Delta\mathbf{W^h}_t, \mathbf{W^h}_t\rangle + \|\Delta\mathbf{W^h}_t\|^2_{\mathbf{H}^{-1}}\\
&= \|\mathbf{W^h}_t\|^2_{\mathbf{H}^{-1}} - 2\eta(\mathrm{sign}(\mathbf{W^h}_t) - \mathbf{W}^*)^T\mathbf{W^h}_t + \|\Delta\mathbf{W^h}_t\|^2_{\mathbf{H}^{-1}}\\
&= \|\mathbf{W^h}_t\|^2_{\mathbf{H}^{-1}} - 2\eta(\mathrm{sign}(\mathbf{W^h}_t) - \mathbf{W}^*)^T\mathbf{W^h}_t + \|\Delta\mathbf{W^h}_t\|^2_{\mathbf{H}^{-1}},
\end{aligned}
$$

so that :

$$
\begin{aligned}
&\|\mathbf{W^h}_{t+1}\|^2_{\mathbf{H}^{-1}} - \|\mathbf{W^h}_t\|^2_{\mathbf{H}^{-1}} \leq 0\\
&\Leftrightarrow 2(\mathrm{sign}(\mathbf{W^h}_t) - \mathbf{W}^*)^T \cdot \mathbf{W^h}_t \geq \|\Delta\mathbf{W^h}_t\|^2_{\mathbf{H}^{-1}}.
\end{aligned}
\tag{11}
$$

We want to show that if $\mathbf{W^h}_t$ is large enough in norm $\|\cdot\|_{\mathbf{H}^{-1}}$, Eq. (11) will be met. First note that, because the dimension is finite there exist two constants $\alpha > 0$ and $\beta > 0$ such that $\forall \mathbf{x} \in \mathbb{R}^d$,

$$
\alpha\|\mathbf{x}\|_{\mathbf{H}^{-1}} < \|\mathbf{x}\|_\infty < \beta\|\mathbf{x}\|_{\mathbf{H}^{-1}}
$$

and also that:

$$
\|\Delta\mathbf{W^h}_t\|^2_{\mathbf{H}^{-1}} = \eta^2\|\mathrm{sign}(\mathbf{W^h}_t) - \mathbf{W}^*\|^2_{\mathbf{H}}.
$$

Then, by triangular inequality:

$$
\eta\|\mathrm{sign}(\mathbf{W^h}_t) - \mathbf{W}^*\|_{\mathbf{H}} \leq \eta(\|\mathrm{sign}(\mathbf{W^h}_t)\|_{\mathbf{H}} + \|\mathbf{W}^*\|_{\mathbf{H}}).
$$

Denoting $(\mathbf{e}_\alpha)_\alpha$ and $(\lambda_\alpha)_\alpha$ the eigenbasis of $\mathbf{H}$ and their associated eigenvalues, we have by Cauchy Schwarz inequality:

$$
\begin{aligned}
\|\mathrm{sign}(\mathbf{W^h}_t)\|^2_{\mathbf{H}} &= \langle \mathbf{H}\cdot\mathrm{sign}(\mathbf{W^h}_t), \mathrm{sign}(\mathbf{W^h}_t)\rangle\\
&= \sum_{\alpha=1}^{d} \lambda_\alpha |\langle \mathrm{sign}(\mathbf{W^h}_t), \mathbf{e}_\alpha\rangle|^2\\
&\leq \sum_{\alpha=1}^{d} \lambda_\alpha \underbrace{\|\mathrm{sign}(\mathbf{W^h}_t)\|_2^2}_{=d} \cdot \underbrace{\|\mathbf{e}_\alpha\|_2^2}_{=1}\\
&\leq d^2\lambda_{\alpha,max},
\end{aligned}
$$

so that:

$$
\|\Delta\mathbf{W^h}_t\|_{\mathbf{H}^{-1}} \leq \eta(d\sqrt{\lambda_{\alpha,max}} + \|\mathbf{W}^*\|_{\mathbf{H}}).
\tag{12}
$$

Thus the right hand side of Eq. 11 is bounded. Also note that:

$$
\begin{aligned}
2(\text{sign}(\mathbf{W^h}_t) - \mathbf{W}^*)^T \cdot \mathbf{W^h}_t &= 2\sum_{i=1}^{d}(1-\text{sign}(W^{\text{h}}_{i,t})W^*_i)|W^{\text{h}}_{i,t}| \\
&\geq 2\sum_{i=1}^{d}(1-|W^*_i|)|W^{\text{h}}_{i,t}| \\
&\geq 2(1-\|\mathbf{W}^*\|_\infty)\sum_{i=1}^{d}|W^{\text{h}}_{i,t}| \\
&\geq 2(1-\|\mathbf{W}^*\|_\infty)\cdot\|\mathbf{W^h}_t\|_\infty,
\end{aligned}
$$

So far we have shown that the left hand side of Eq.11 is lower bounded by a constant ($\neq 0$) times the infinite norm of $\mathbf{W^h}_t$, while the right hand side is bounded. Therefore to ensure Eq. (11) it suffices that:

$$
\begin{aligned}
&2(1-\|\mathbf{W}^*\|_\infty)\cdot\|\mathbf{W^h}_t\|_\infty \geq \eta(d\sqrt{\lambda_{\alpha,max}} + \|\mathbf{W}^*\|_{\mathbf{H}}) \\
&\Leftrightarrow \|\mathbf{W^h}_t\|_\infty \geq \frac{\eta(d\sqrt{\lambda_{\alpha,max}} + \|\mathbf{W}^*\|_{\mathbf{H}})}{2(1-\|\mathbf{W}^*\|_\infty)}.
\end{aligned}
$$

And thus to ensure Eq. (11) it suffices that:

$$
\|\mathbf{W^h}_t\|_{\mathbf{H}^{-1}} \geq \frac{\eta(d\sqrt{\lambda_{\alpha,max}} + \|\mathbf{W}^*\|_{\mathbf{H}})}{2\alpha(1-\|\mathbf{W}^*\|_\infty)}.
$$

Denoting $M = \frac{\eta(d\sqrt{\lambda_{\alpha,max}} + \|\mathbf{W}^*\|_{\mathbf{H}})}{2\alpha(1-\|\mathbf{W}^*\|_\infty)}$, we can conclude that $\|\mathbf{W^h}_t\|_{\mathbf{H}^{-1}} \geq M \Rightarrow \|\mathbf{W^h}_{t+1}\|^2_{\mathbf{H}^{-1}} < \|\mathbf{W^h}_t\|^2_{\mathbf{H}^{-1}}$. And because the update $\Delta\mathbf{W^h}_t$ is bounded in norm $\|\cdot\|_{\mathbf{H}^{-1}}$, an absolute upper bound of $\mathbf{W^h}_t$ is :

$$
C = \beta\max(\|\mathbf{W^h}_0\|_{\mathbf{H}^{-1}},\ M + \eta(d\sqrt{\lambda_{\alpha,max}} + \|\mathbf{W}^*\|_{\mathbf{H}})).
$$

Thus we have proven that $\mathbf{W}^* \in \mathscr{B}_\infty \Rightarrow \exists C > 0, \forall t \in \mathbb{N}, \|\mathbf{W^h}_t\|_\infty < C$

□

Lemma 2 (hidden Weight Trajectory)*:* Let $\mathbf{W^h}$ optimize a quadratic binary task according to the dynamics $\mathbf{W^h}_{t+1} = \mathbf{W^h}_t - \eta\mathbf{H}(\text{sign}(\mathbf{W^h}_t) - \mathbf{W}^*)$ and assume $\mathbf{H} = \text{diag}(\lambda_1, \ldots \lambda_d)$. Then:

$$
|W^*_i| > 1 \implies W^{\text{h}}_{i,t} \sim_{t\to+\infty} \underbrace{\text{sign}(W^*_i)\eta\lambda_i(|W^*_i|-1)t}_{=\widetilde{W^{\text{h}}_i}} \tag{13}
$$

Proof of Lemma 2. If $\mathbf{H} = \text{diag}(\lambda_1, \ldots \lambda_d)$, the dynamics of $\mathbf{W^h}_t$ defined in Eq. (5) simply rewrites component-wise:

$$
\forall i \in [\![1,d]\!]\ ,\ \Delta W^{\text{h}}_{i,t} = W^{\text{h}}_{i,t+1} - W^{\text{h}}_{i,t} = -\eta\lambda_i(\text{sign}(W^{\text{h}}_{i,t}) - W^*_i). \tag{14}
$$

By Lemma 1, components W_i such that $|W^*_i| < 1$ are bounded.

For components i where $|W_i^*| > 1$, $\Delta W_{i,t}^{\mathrm{h}}$ has the sign of W_i^* since Eq. (14) rewrites:

$$\Delta W_{i,t}^{\mathrm{h}} = \mathrm{sign}(W_i^*)\underbrace{\eta\lambda_i(|W_i^*| - \mathrm{sign}(W_i^* W_{i,t}^{\mathrm{h}}))}_{>0}, \tag{15}$$

so that $W_{i,t}^{\mathrm{h}}$ necessarily ends up having the same sign as W_i^*, hence there exists $t_{0,i} \in \mathbb{N}$ such that :

$$\forall t > t_{0,i}, \quad \Delta W_{i,t}^{\mathrm{h}} = \mathrm{sign}(W_i^*)\eta\lambda_i(|W_i^*| - 1). \tag{16}$$

By definition of $t_{0,i}$, $W_{i,t}^{\mathrm{h}}$ and W_i^* have opposite sign before $t_{0,i}$ so that:

$$\forall t \leq t_{0,i}, \quad \Delta W_{i,t}^{\mathrm{h}} = \mathrm{sign}(W_i^*)\eta\lambda_i(1 + |W_i^*|). \tag{17}$$

Therefore, summing Eq. (14) between 0 and t yields :

$$\begin{aligned} W_{i,t}^{\mathrm{h}} &= W_{i,0}^{\mathrm{h}} + \sum_{u=0}^{t_{0,i}} \mathrm{sign}(W_i^*)\eta\lambda_i(|W_i^*| + 1) \\ &\quad + \sum_{u=t_{0,i}+1}^{t} \mathrm{sign}(W_i^*)\eta\lambda_i(|W_i^*| - 1) \\ &= W_{i,0}^{\mathrm{h}} + \mathrm{sign}(W_i^*)\eta\lambda_i(|W_i^*| + 1)t_{0,i} \\ &\quad + \mathrm{sign}(W_i^*)\eta\lambda_i(|W_i^*| - 1)(t - t_{0,i}) \\ &\sim_{t\to+\infty} \underbrace{\mathrm{sign}(W_i^*)\eta\lambda_i(|W_i^*| - 1)}_{=\widetilde{W_i^{\mathrm{h}}}} t \end{aligned} \tag{18}$$

□

Theorem 1 (Importance of hidden Weights in a quadratic binary task)*:* Let $\mathbf{W}$ optimize a quadratic binary task according to the dynamics $\mathbf{W^h}_{t+1} = \mathbf{W^h}_t - \eta\mathbf{H}(\mathrm{sign}(\mathbf{W^h}_t) - \mathbf{W}^*)$ and assume $\mathbf{H} = \mathrm{diag}(\lambda_1, \ldots \lambda_d)$. Then, for any component i such that $|W_i^*| > 1$, the variation of loss resulting from flipping $\mathrm{sign}(W_{i,t}^{\mathrm{h}}) \to -\mathrm{sign}(W_{i,t}^{\mathrm{h}})$ is:

$$\Delta_i \mathscr{L}(\mathbf{W^h}_t) = 2\lambda_i|W_i^*| = 2\left(\lambda_i + \frac{|\widetilde{W_i^{\mathrm{h}}}|}{\eta}\right) + \mathscr{O}(\frac{1}{t}) \tag{19}$$

Proof of Theorem. 1

Proof. Using Eq. (4), the loss reads:

$$\begin{aligned} \mathscr{L}(\mathbf{W^h}_t) &= \frac{1}{2}(\mathrm{sign}(\mathbf{W^h}_t) - \mathbf{W}^*)^T\mathbf{H}(\mathrm{sign}(\mathbf{W^h}_t) - \mathbf{W}^*) \\ &= \frac{1}{2}\sum_{i=1}^{n} \lambda_i(\mathrm{sign}(W_{i,t}^{\mathrm{h}}) - W_i^*)^2 \\ &= \frac{1}{2}\sum_{i,|W_i^*|\leq 1} \lambda_i(\mathrm{sign}(W_{i,t}^{\mathrm{h}}) - W_i^*)^2 \\ &\quad + \frac{1}{2}\sum_{i,|W_i^*|>1} \lambda_i(\mathrm{sign}(W_{i,t}^{\mathrm{h}}) - W_i^*)^2. \end{aligned}$$

Using Lemma 2, for all components i such that $|W_i^*| > 1$, there exists $t_{0,i}$ such that for all $t > t_{0,i}$, $\text{sign}(W_{i,t}^{\text{h}}) = \text{sign}(W_i^*)$ and therefore $\frac{1}{2}\lambda_i(\text{sign}(W_{i,t}^{\text{h}}) - W_i^*)^2 = \frac{1}{2}\lambda_i(1-|W_i^*|)^2$. Defining $T = \max_{i||W_i^*|>1}(t_{0,i})$, the loss rewrites for $t > T$:

$$\begin{aligned}\mathscr{L}(\mathbf{W^h}_t) = &\frac{1}{2}\sum_{i,|W_i^*|\leq 1}\lambda_i(\text{sign}(W_{i,t}^{\text{h}}) - W_i^*)^2\\ &+\frac{1}{2}\sum_{i,|W_i^*|>1}\lambda_i(|W_i^*|-1)^2\end{aligned}$$

Then, the increase in energy if a binary component in the $|W_i^*| > 1$ sum is switched is :

$$\Delta_i\mathscr{L}(\mathbf{W^h}_t) = \frac{\lambda_i}{2}((|W_i^*|+1)^2 - (|W_i^*|-1)^2) = 2\lambda_i|W_i^*| \tag{20}$$

Using the explicit form of $W_{i,t}^{\text{h}}$ in Eq. (18) along with Eq. (20), we get:

$$\begin{aligned}W_{i,t}^{\text{h}} &= W_{i,0}^{\text{h}} + \text{sign}(W_i^*)\eta\lambda_i(|W_i^*|+1)t_{0,i}\\ &\quad + \text{sign}(W_i^*)\eta\lambda_i(|W_i^*|-1)(t-t_{0,i})\\ &= W_{i,0}^{\text{h}} + \text{sign}(W_i^*)\eta\lambda_i\left(\frac{\Delta_i\mathscr{L}}{2\lambda_i}+1\right)t_{0,i}\\ &\quad + \text{sign}(W_i^*)\eta\lambda_i\left(\frac{\Delta_i\mathscr{L}}{2\lambda_i}-1\right)(t-t_{0,i})\\ &= W_{i,0}^{\text{h}} + \text{sign}(W_i^*)\eta\frac{\Delta_i\mathscr{L}}{2}t + \text{sign}(W_i^*)\eta\lambda_i(2t_{0,i}-t)\\ &= \text{sign}(W_i^*)\eta\left(\frac{\Delta_i\mathscr{L}}{2}-\lambda_i\right)t + W_{i,0}^{\text{h}} + \text{sign}(W_i^*)\eta\lambda_i 2t_{0,i}.\end{aligned}$$

Since $W_{i,t}^{\text{h}}$ has the same sign as W_i^* for t being large enough, multiplying both sides for the last equation and dividing by t yields:

$$\Delta_i\mathscr{L}(\mathbf{W^h}_t) = 2\left(\lambda_i + \frac{\widetilde{|W_i^{\text{h}}|}}{\eta}\right)\underbrace{-2\frac{|W_{i,0}^{\text{h}}| + \eta\lambda_i 2t_{0,i}}{\eta t}}_{=\mathscr{O}(\frac{1}{t})} \tag{21}$$

□

Supplementary Note 6: Comparison Between the Hidden Weights of Binarized Neural Networks and the Weights of Full Precision Networks

In this supplementary note, we illustrate on a 2-D optimization task how hidden weights in a binarized model differ from the usual full precision weights, and why the former are good candidates for synaptic consolidation. The color map of Suppl. Fig. 3 denotes a 2-D landscape where the darker the color, the higher the cost. The global minimum is denoted in red by $\mathbf{W}^*$. The binarized model in Supp. Fig. 3a has two parameters given by the sign of two hidden weights W_x^{h} and W_y^{h}. The binarized model can thus be in four different states given by the corners of the unit sphere for the infinite norm $\mathscr{B}_\infty$. If we consider a case where $\mathbf{W}^*$ is not a corner of $\mathscr{B}_\infty$, the binarized model cannot converge. Instead, hidden weights keep being updated by gradients evaluated in the binarized parameters. We show in Supplementary Note 5 that the vector $\mathbf{W^h}$ will diverge if $\mathbf{W}^*$ is outside $\mathscr{B}_\infty$, even if we increase the dimension of the problem. We intuitively see in Suppl. Fig. 3a that because W_y^* is between -1 and 1, and $W_x^* > 1$, the binarized value of W_x^{h} is more important than the binarized value of W_y^{h} with respect to optimization. On the other hand, Suppl. Fig. 3b shows the same optimization problem solved by a full precision model. The model converges to $\mathbf{W}^*$ and contrary to the binarized case, the knowledge of the final state of W_x and W_y cannot be leveraged to learn a second task.

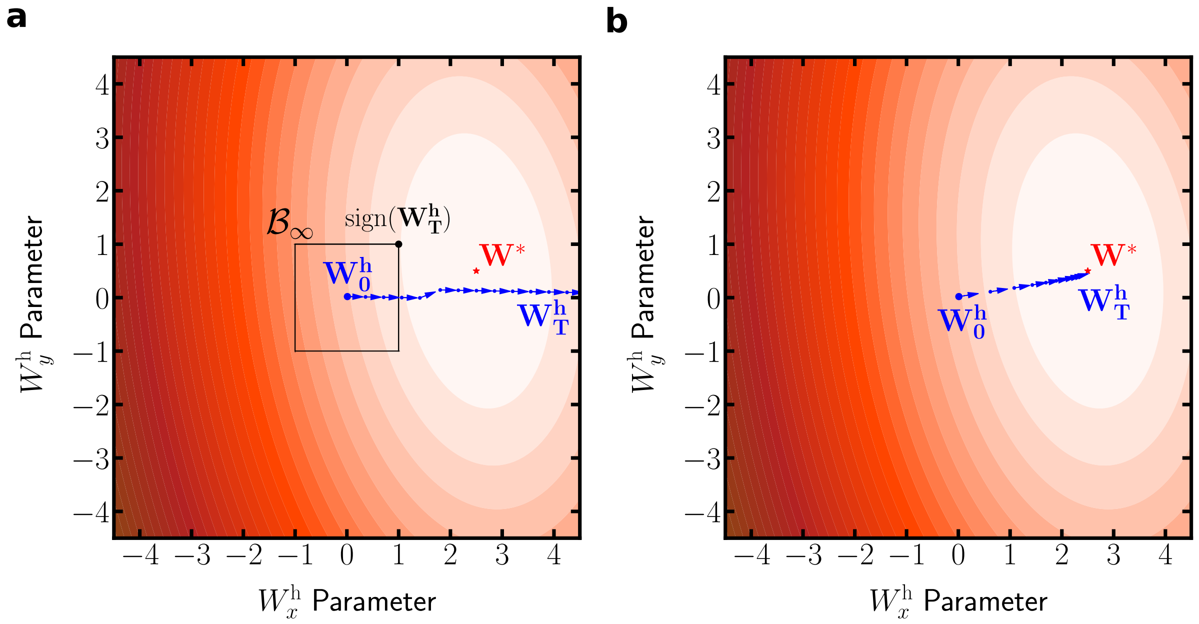

Supplementary Figure 3. Comparison between a binarized model hidden weights and full precision weights. Weights trajectories in the case of a 2-D optimization task. The hidden weights of a binary model **a** are an accumulation of gradients evaluated in the binary values (black dot). **b** In the case of a full precision model, the weight values cannot directly be used for weight consolidation.

Supplementary Note 7: Learning Rate Decay

In this supplementary note, we investigate the performance of a learning rate decay scheduler to see how it compares to our metaplastic binarized neural network approach. We study the setting of learning six permuted MNISTs and investigate learning rate schedulers where the learning rate is divided by a constant factor between each task. We list in Table 4 the performance for several values of initial learning rates and dividing factors. For instance, an initial learning rate of 10^{-2} and dividing factor of 10 means that the six tasks are learned respectively with the learning rates : 10^{-2}, 10^{-3}, 10^{-4}, 10^{-5}, 10^{-6}, and 10^{-7}.

(initial LR, dividing factor)	Task 1	Task 2	Task 3	Task 4	Task 5	Task 6
(0.001, 5.0)	60.93(9.5)	74.49(6.2)	73.28(7.1)	63.47(2.6)	60.54(3.9)	95.71(0.2)
(0.01, 5.0)	27.01(5.8)	51.15(7.2)	79.86(4.5)	86.53(0.9)	81.45(4.1)	96.62(0.1)
(0.1, 5.0)	9.70(0.5)	11.94(2.4)	11.50(3.1)	18.26(11.3)	72.47(14.4)	96.98(0.2)
(0.001, 10.0)	84.98(6.5)	86.02(3.3)	62.58(8.7)	58.86(5.0)	82.77(1.7)	91.42(0.2)
(0.01, 10.0)	57.21(8.2)	86.35(3.9)	84.13(2.5)	67.00(4.6)	75.12(5.7)	94.77(0.2)
(0.1, 10.0)	10.58(1.0)	14.49(7.8)	11.64(2.8)	28.51(24.5)	66.67(26.5)	95.88(0.2)
(0.001, 20.0)	**93.23(2.4)**	**86.85(1.3)**	**73.37(1.8)**	**82.83(3.6)**	**87.76(0.7)**	**73.29(2.3)**
(0.01, 20.0)	**89.28(1.4)**	**93.40(1.3)**	**80.13(5.0)**	**76.71(7.4)**	**88.67(0.9)**	**82.15(1.0)**
(0.1, 20.0)	10.16(0.7)	13.16(4.1)	16.46(3.4)	40.85(31.0)	41.88(43.6)	88.77(0.4)
(0.005, 5.0)	38.38(3.0)	65.13(5.5)	78.35(6.8)	82.42(2.0)	77.61(5.2)	96.56(0.0)
(0.05, 5.0)	9.90(0.1)	11.60(1.6)	11.03(3.5)	24.04(16.6)	69.18(13.6)	96.93(0.1)
(0.5, 5.0)	10.03(0.1)	10.04(0.3)	11.29(2.0)	12.73(6.6)	28.34(15.0)	97.12(0.1)
(0.005, 10.0)	71.14(6.4)	87.20(2.6)	86.06(2.8)	63.66(5.6)	75.13(2.5)	93.91(0.1)
(0.05, 10.0)	10.14(0.1)	10.58(1.7)	9.79(0.5)	13.72(3.4)	51.86(34.4)	95.58(0.2)
(0.5, 10.0)	10.09(0.3)	9.99(0.2)	9.74(0.5)	10.14(0.5)	38.81(22.7)	96.79(0.2)
(0.005, 20.0)	**91.51(2.1)**	**93.67(1.1)**	**75.41(6.1)**	**80.62(1.3)**	**88.69(0.4)**	**80.89(0.9)**
(0.05, 20.0)	10.01(1.2)	9.90(1.3)	10.08(0.8)	39.40(37.4)	71.63(34.5)	85.22(1.3)
(0.5, 20.0)	10.32(0.7)	10.27(1.1)	9.89(0.2)	15.57(4.0)	14.13(4.6)	91.93(0.5)

Supplementary Table 4. Permuted MNIST experiment with learning rate decay. The accuracy for each task is averaged over five runs and standard deviation is given between parenthesis. Best settings are in bold font.

Supplementary Note 8: Increasing Synapse Complexity for Steady-State Continual Learning

In this supplementary note, we show that one limitation of the metaplasticity model presented in the main article can be alleviated by considering a more complex synaptic model inspired by the metaplasticity model of Benna and Fusi [5]. The metaplasticity model presented in the main article has an aging property that prevents it from learning new tasks after learning a finite number of tasks (depending on the capacity of the network). For instance, Supplementary Figure 4(b) shows the accuracies of ten tasks learned by the metaplasticity model introduced in the main article. While the network successfully learns up to seven tasks, the last three tasks are not properly learned because all the weights have been consolidated. The type of continual learning achieved by this network is therefore non steady-state, consistently with much of the machine learning literature on continual learning[6], but unlike the brain.

The metaplasticity model introduced in [5] describes synapses with several hidden variables interacting over a wide range of timescales through diffusion processes. The slowest variable features a leakage term, allowing the possibility to reach a steady-state type of consolidated learning, where the newest memories can replace the firstly trained ones. Here, we propose training binarized neural networks with synapses featuring not a simple hidden weight, but a collection of them interacting over a wide range of timescales in a way inspired by [5]. This approach can have several benefits. First, the hidden weights tend to evolve stochastically in conventional binarized neural networks due to the stochastic nature of data batches. This means that in our original metaplasticity approach, if a hidden weight gets carried too far away from zero because of the noise, it will be consolidated. The more complicated synapses inspired by [5] can provide a cleaner signal to perform weight consolidation and constitute promising candidates to solve the issue of noise-induced consolidation. Second, and more importantly, thanks to the leakage on the slower variable, we hope to provide the binarized neural network with a truly steady-state form of continual learning.

In our model, each synapse features four hidden variables (W_1^{h}, W_2^{h}, W_3^{h}, and W_4^{h}), which evolve according to :

$$\begin{cases} W_1^{\mathrm{h}}(t+1) = W_1^{\mathrm{h}}(t) - \eta \dfrac{\partial \mathscr{L}}{\partial W_{\mathrm{b}}} + g_{1,2}(W_2^{\mathrm{h}}(t) - W_1^{\mathrm{h}}(t)) \quad \text{if} \quad (W_1^{\mathrm{h}}(t) - W_4^{\mathrm{h}}(t)) \cdot \mathrm{sign}(W_4^{\mathrm{h}}(t)) > 0 \\ W_1^{\mathrm{h}}(t+1) = W_1^{\mathrm{h}}(t) - \eta \dfrac{\partial \mathscr{L}}{\partial W_{\mathrm{b}}} + g_{1,2}(W_2^{\mathrm{h}}(t) - W_1^{\mathrm{h}}(t)) + \alpha(W_4^{\mathrm{h}}(t) - W_1^{\mathrm{h}}(t)) \quad \text{otherwise} \\ W_2^{\mathrm{h}}(t+1) = W_2^{\mathrm{h}}(t) + g_{1,2}(W_1^{\mathrm{h}}(t) - W_2^{\mathrm{h}}(t)) + g_{2,3}(W_3^{\mathrm{h}}(t) - W_2^{\mathrm{h}}(t)) \\ W_3^{\mathrm{h}}(t+1) = W_3^{\mathrm{h}}(t) + g_{2,3}(W_2^{\mathrm{h}}(t) - W_3^{\mathrm{h}}(t)) + g_{3,4}(W_4^{\mathrm{h}}(t) - W_3^{\mathrm{h}}(t)) \\ W_4^{\mathrm{h}}(t+1) = W_4^{\mathrm{h}}(t) + g_{3,4}(W_3^{\mathrm{h}}(t) - W_4^{\mathrm{h}}(t)) - \varepsilon W_4^{\mathrm{h}}(t) \quad \text{if} \quad |W_3^{\mathrm{h}}(t)| > |W_4^{\mathrm{h}}(t)| \\ W_4^{\mathrm{h}}(t+1) = W_4^{\mathrm{h}}(t) + g_{3,4} \cdot f_{\mathrm{meta}}(W_4^{\mathrm{h}})(W_3^{\mathrm{h}}(t) - W_4^{\mathrm{h}}(t)) - \varepsilon W_4^{\mathrm{h}}(t) \quad \text{otherwise} \end{cases} \tag{22}$$

The typical evolution of those hidden variables is described in Supplementary Figure 4(a) and is plotted in Supplementary Figure 4(d). The hidden variables evolve over a wide range of timescales through a diffusion chain process. The deepest variable W_4^{h} is a slower and smoother version of W_1^{h}, which is thus relevant for consolidation. These equations are analogous to the ones used in[5], with two additions. The addition of a f_{meta} factor in the last equation consolidates further the slowest variable W_4^{h}. Additionally, we introduce a direct feedback from W_4^{h} to W_1^{h} when W_1^{h} is smaller than W_4^{h} in absolute value or opposite sign, because we found that feedback through the path involving the intermediary variables was too slow to induce proper memory effects. The effect of the direct feedback is shown in Figure 4(d) after 10^5 iterations. It forces a consolidated weight to be unconsolidated on a timescale governed by the ε decay term on W_4^{h}. We use $\eta = 5 \cdot 10^{-3}$, $g_{1,2} = 10^{-2}$, $g_{2,3} = 10^{-3}$, $g_{3,4} = 10^{-4}$, $\varepsilon = 3 \cdot 10^{-5}$, $\alpha = 5 \cdot 10^{-3}$ and the same f_{meta} as in the main article with $m = 10.0$.

These two additions are necessary as the dynamics of the synapses differ substantially when training binarized neural networks from the situation of [5]. In [5], synaptic updates occur following randomly presented patterns, in an independent and identically distributed fashion. Our continual learning situation is different, because there are two distinct timescales at play: a short timescale constituted by the training iterations within one task, and a long timescale constituted by the different tasks. The slowest variable evolves slowly at the intra-task timescale

but rapidly with respect to the long timescale. We introduce f_{meta} to accommodate for this timescale asymmetry. Another difference comes from the sequential synaptic updates, which follow the gradient of a loss function and are therefore highly correlated on shorter time scales. For this reason, the influence of the slowest variable on W_1^{h} through the diffusion chain cannot effectively protect from the correlated gradients of the new task. We thus add a unidirectional feedback connection parameterized by α (Suppl. Fig. 4 (a)) between the slowest variable and W_1^{h} to provide better consolidation. The two modifications of the model allow W_4^{h} to be more stable on the longer timescales of our setup, while allowing to W_1^{h} react on its shorter ones.

Our results, presented in Supplementary Figure 4(c), and discussed in the main article, show that binarized neural networks featuring such complex synapses can learn tasks sequentially similarly to our simpler synapse model, and in addition, new tasks can be learned while older tasks are gradually forgotten. The histograms of hidden variables (Supplementary Figure 4(e)) also evidence that weights do not accumulate to high values.

We then show in Suppl. Fig. 5(a) how the model performs when a sequence of 20 tasks is learned. In this situation, the system reaches a “true” steady state. This is observed by plotting the distributions of the hidden variables in Suppl. Fig. 5(b), superimposed over the three most recent tasks. We find that the capacity of the model in this true steady state regime is reduced compared to the more transient regime observed during the first ten tasks in Suppl. Fig. 4, as the accuracy of the last learned tasks drops more rapidly in Suppl. Fig. 5(a) than in Suppl. Fig. 4(c). This result is in accordance with the literature on this type of truly steady-state learning [5, 7, 8].

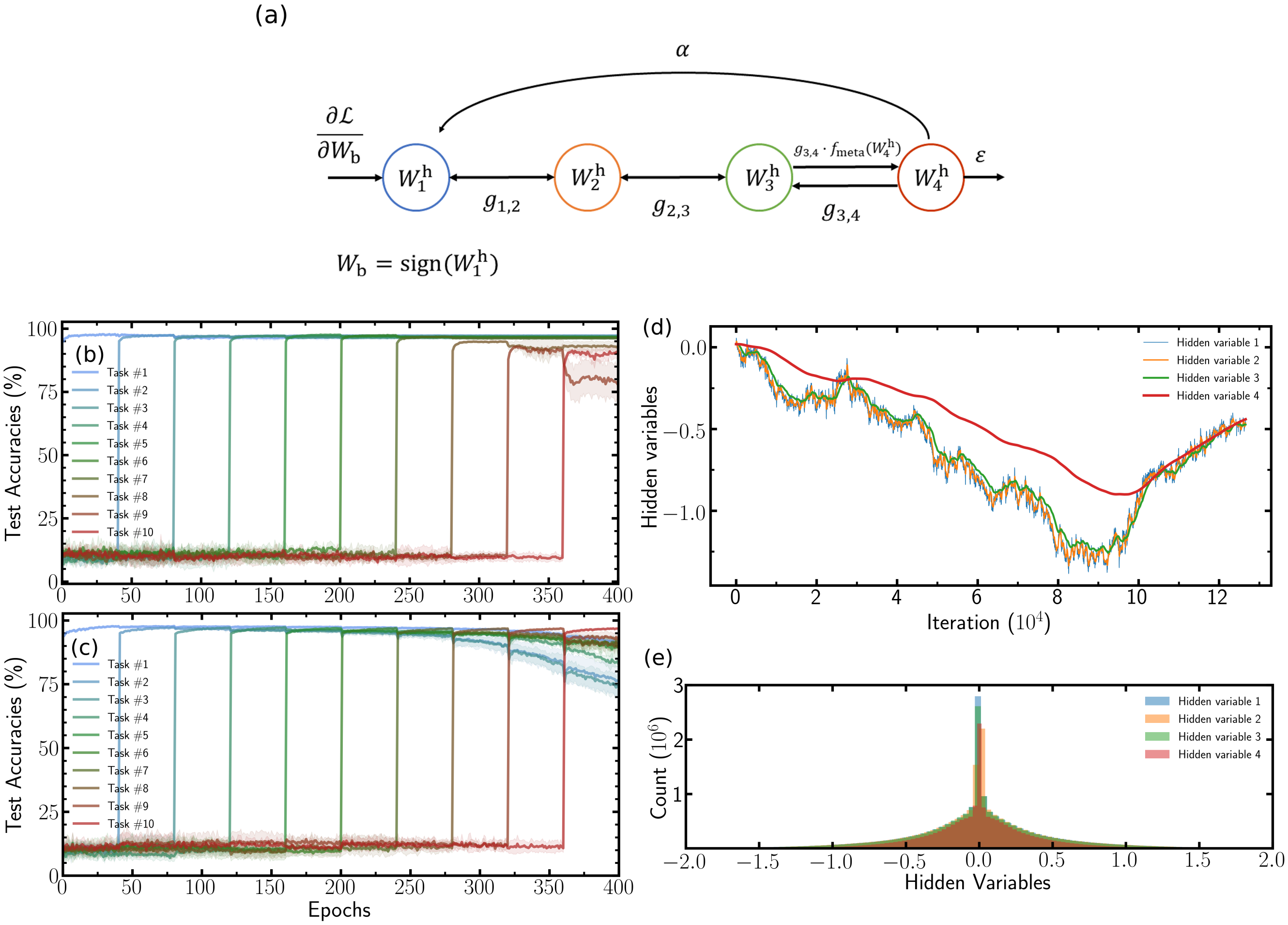

Supplementary Figure 4. **Complex synapse model (a)** Schematic of a more complex synapse model. **(b)** Test accuracies of ten tasks for a metaplastic BNN as introduced in our main article with $m = 1.35$ and two hidden layers of 4,096 units. The tasks are learned until no further learning can be done (task #8 to #10 are not properly learned). **(c)** Same architecture but with our new algorithm with four hidden variables. The model is still able to learn several tasks sequentially but older tasks are gradually forgotten and new tasks can always be learned. The curves are averaged over five runs and shadows stand for one standard deviation. **(d)** Trajectories of the hidden variables as a function of training iterations. The deeper the hidden variable, the slower and smoother it behaves, providing a cleaner signal for consolidation. **(e)** Distribution of the hidden variables after learning 10 tasks. Unlike the distribution presented in the body text, hidden weights do not accumulate to ever increasing values and new tasks can always be learned.

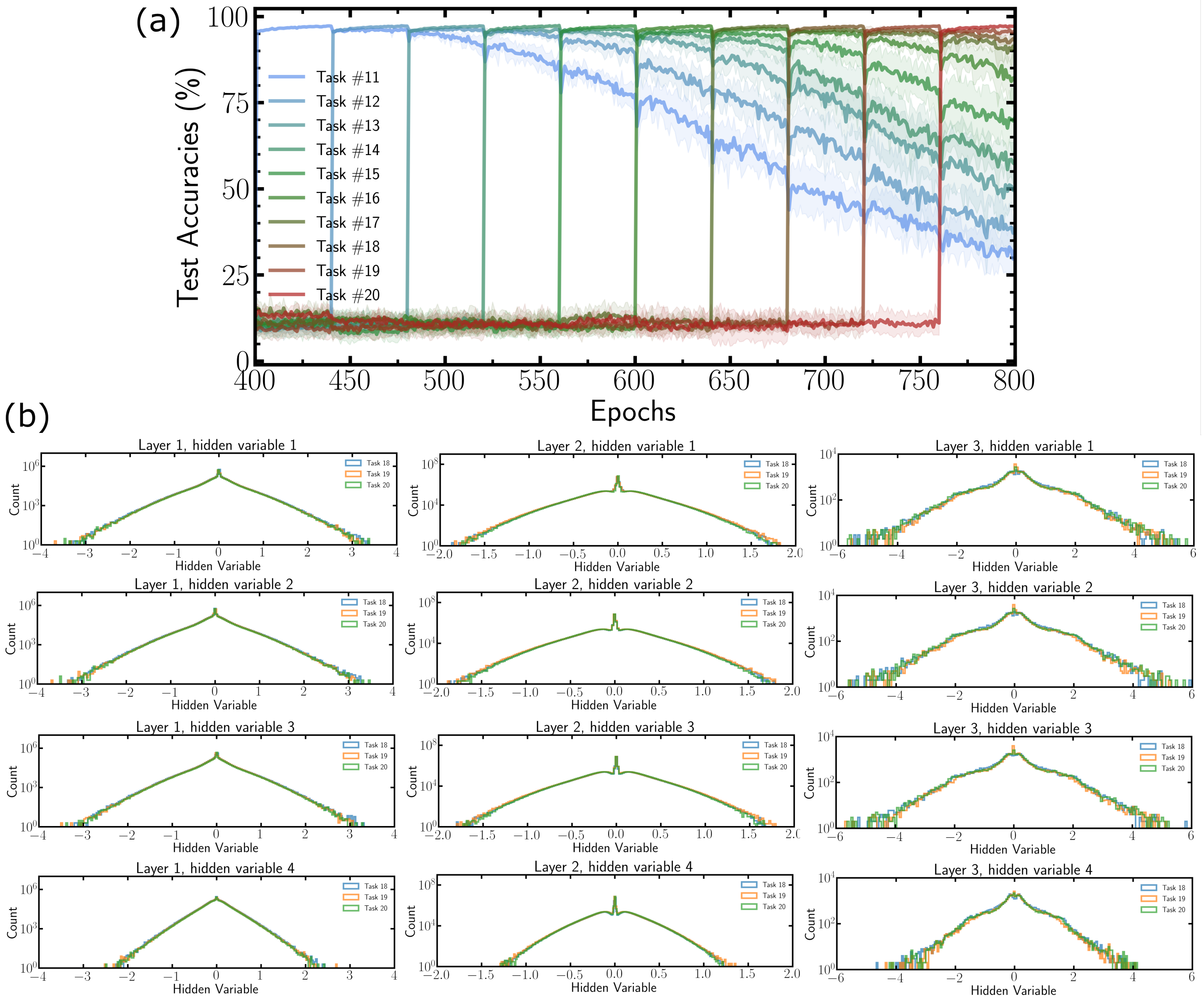

Supplementary Figure 5. Steady-state regime (a) Test accuracy on the ten most recent tasks when learning a long sequence of tasks. The stationary regime exhibit graceful forgetting where oldest tasks are forgotten and new tasks can always be learned. The curves are averaged over five runs and shadows stand for one standard deviation. (b) Distribution of hidden variables for each layer (horizontally) and each hidden variable (vertically). Distributions are superimposed over the three most recent tasks. We observe that the steady state have been reached.

Finally, we investigate the impact of removing the feedback process linking the slowest hidden variable to the first one, in multiple situations. We let the hidden variables evolve only through the main connections and remove the feedback process: we set $\alpha = 0$ and $f_{\text{meta}} = 1$ in Suppl. Fig. 4(a). The results are listed in the Supplementary Table 5 for 21 values of the parameters of the synapses, covering cases with more hidden variables and/or slower time scales. In all these situations, we observe some memory signal for Tasks 8 and 9, with varying accuracy depending on the parameter choice. However, the accuracy of Task 7 is always back to near-random guess, suggesting that catastrophic forgetting remains strong in the absence of our model modifications. This result is consistent with our interpretation that the influence of the slowest (last) hidden variable over the fastest one through the main connections is too weak to protect the first variable from the strongly correlated gradients related to the current task.

Hidden Variables number	Parameters $g_{i,i+1}$, ε	Task 7 Test Acc. (%)	Task 8 Test Acc. (%)	Task 9 Test Acc. (%)	Task 10 Test Acc. (%)
4	10^{-2}, 10^{-3},10^{-4},10^{-5}	10.44	14.86	31.1	97.57
	$2\cdot10^{-2}$, $2\cdot10^{-3}$, $2\cdot10^{-4}$, $2\cdot10^{-5}$	6.92	15.6	37.53	97.77
	$3\cdot10^{-2}$, $3\cdot10^{-3}$, $3\cdot10^{-4}$, $3\cdot10^{-5}$	13.26	16.56	36.73	95.76
	$4\cdot10^{-2}$, $4\cdot10^{-3}$, $4\cdot10^{-4}$, $4\cdot10^{-5}$	11.02	11.31	40.08	97.52
	$7\cdot10^{-2}$, $7\cdot10^{-3}$, $7\cdot10^{-4}$, $7\cdot10^{-5}$	11.0	13.77	31.25	97.07
	$9\cdot10^{-2}$, $9\cdot10^{-3}$, $9\cdot10^{-4}$, $9\cdot10^{-5}$	8.57	12.94	23.42	96.01
	10^{-1}, 10^{-2},10^{-3},10^{-4}	11.06	6.42	31.4	96.99
5	10^{-2}, 10^{-3}, 10^{-4}, 10^{-5}, 10^{-6}	10.1	21.21	45.19	97.49
	$2\cdot10^{-2}$, $2\cdot10^{-3}$, $2\cdot10^{-4}$, $2\cdot10^{-5}$, $2\cdot10^{-6}$	12.94	12.72	48.04	97.61
	$3\cdot10^{-2}$, $3\cdot10^{-3}$, $3\cdot10^{-4}$, $3\cdot10^{-5}$, $3\cdot10^{-6}$	9.62	14.03	35.06	96.79
	$4\cdot10^{-2}$, $4\cdot10^{-3}$, $4\cdot10^{-4}$, $4\cdot10^{-5}$, $4\cdot10^{-6}$	17.29	16.49	42.48	97.5
	$7\cdot10^{-2}$, $7\cdot10^{-3}$, $7\cdot10^{-4}$, $7\cdot10^{-5}$, $7\cdot10^{-6}$	10.06	13.86	38.81	96.7
	$9\cdot10^{-2}$, $9\cdot10^{-3}$, $9\cdot10^{-4}$, $9\cdot10^{-5}$, $9\cdot10^{-6}$	15.16	15.23	45.78	97.07
	10^{-1}, 10^{-2}, 10^{-3}, 10^{-4}, 10^{-5}	10.05	18.86	38.99	97.13
6	10^{-2}, 10^{-3}, 10^{-4}, 10^{-5}, 10^{-6}, 10^{-7}	7.17	13.07	31.76	96.35
	$2\cdot10^{-2}$, $2\cdot10^{-3}$, $2\cdot10^{-4}$, $2\cdot10^{-5}$, $2\cdot10^{-6}$, $2\cdot10^{-7}$	11.00	18.91	35.88	96.93
	$3\cdot10^{-2}$, $3\cdot10^{-3}$, $3\cdot10^{-4}$, $3\cdot10^{-5}$, $3\cdot10^{-6}$, $3\cdot10^{-7}$	13.09	18.05	45.85	97.44
	$4\cdot10^{-2}$, $4\cdot10^{-3}$, $4\cdot10^{-4}$, $4\cdot10^{-5}$, $4\cdot10^{-6}$, $4\cdot10^{-7}$	11.45	17.47	44.48	97.53
	$7\cdot10^{-2}$, $7\cdot10^{-3}$, $7\cdot10^{-4}$, $7\cdot10^{-5}$, $7\cdot10^{-6}$, $7\cdot10^{-7}$	11.15	17.08	58.89	97.72
	$9\cdot10^{-2}$, $9\cdot10^{-3}$, $9\cdot10^{-4}$, $9\cdot10^{-5}$, $9\cdot10^{-6}$, $9\cdot10^{-7}$	9.54	16.29	42.53	96.75
	10^{-1}, 10^{-2}, 10^{-3}, 10^{-4}, 10^{-5}, 10^{-6}	8.7	14.87	44.74	97.6

Supplementary Table 5. Control experiment to verify that the feedback process we introduce on the slowest hidden variable is required. The results in this table correspond to the ten permuted MNISTs experiment with hidden variables evolving through the main connections only (as in [5]) for a wide range of parameters.

Supplementary Note 9: Sequential Training of the MNIST and Fashion-MNIST Datasets

To test the ability of our binarized neural network to learn several tasks sequentially, we train a binarized neural network sequentially on two tasks in a more difficult situation than permuted MNISTs. When learning permuted versions of MNIST, the relevant input features do not overlap extensively between tasks which makes it easier for the network to learn sequentially. For this reason, we now train a binarized neural network with two hidden layers of 4,096 units to learn sequentially the MNIST dataset and the Fashion-MNIST dataset [9] which consists of fashion items images belonging to ten classes. Suppl. Fig. 6(b) shows the result of the training of a $m = 1.5$ binarized neural network, with 50 epochs on MNIST and 50 epochs on Fashion-MNIST (Suppl. Fig. 6(d) shows the reverse training order). Suppl. Figs. 6(a) and (c) also show the result for the conventional binarized neural network ($m = 0$). Baselines define the accuracies the binarized neural network would have obtained had it been trained on each of these tasks separately. The baseline of Fashion-MNIST is taken in Suppl. Fig. 6(a) (orange curve after 100 epochs) and the baseline of MNIST in Suppl. Fig. 6(c) (blue curve after 100 epochs). We observe that the metaplastic binarized neural network is able to learn both tasks sequentially with baseline accuracies regardless of the order chosen to learn the tasks.

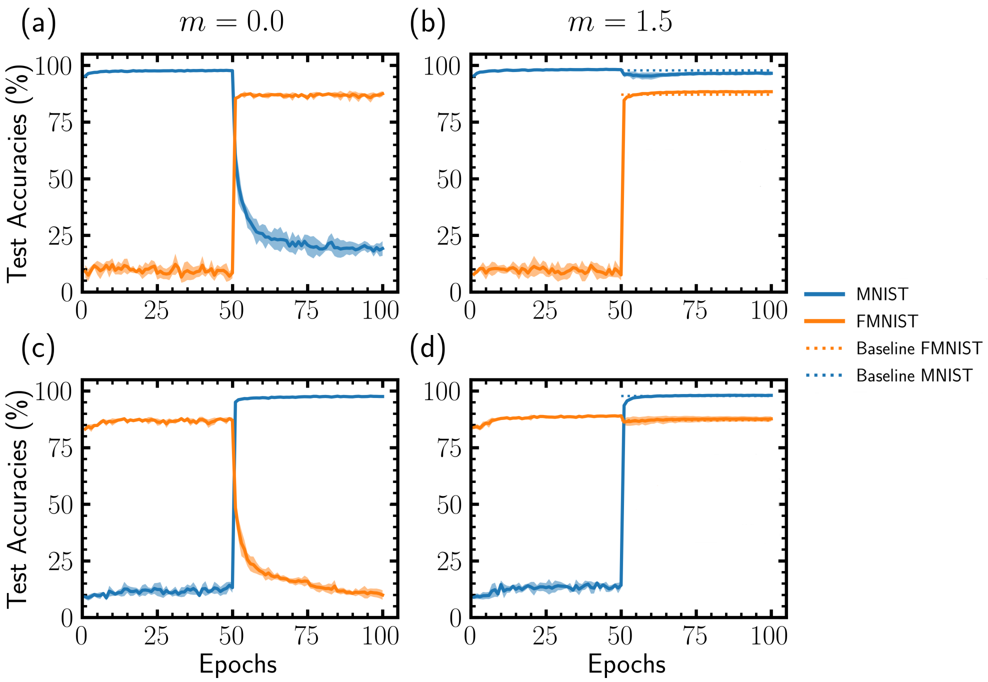

Supplementary Figure 6. MNIST/Fashion-MNIST sequential learning. Binarized neural network learning MNIST and Fashion-MNIST sequentially ((a) and (b)) or Fashion-MNIST and MNIST ((c) and (d)) for two values of the metaplastic parameter m. $m = 0$ corresponds to a conventional BNN ((a) and (c)), $m = 1.5$ is a metaplastic BNN ((b) and (d)). Curves are averaged over five runs and shadows correspond to one standard deviation.

Supplementary Note 10: Sequential Training of the MNIST and USPS Datasets

In this note, we investigate the sequential training of two closely related tasks: the handwritten digits of the MNIST (Supp. Fig. 7(a)) and of the United States Postal Services (USPS, Supp. Fig. 7(b)) datasets. This situation differs from permuted MNIST (Fig. 2 in the main body text), sequential Fashion-MNIST / MNIST (Suppl. Note 9) and incremental CIFAR-10/CIFAR-100 (Suppl. Note 11), where the incrementally trained tasks were always largely uncorrelated in nature.

We compare the accuracy of a metaplastic binarized neural network trained sequentially on MNIST and USPS, with the two networks trained independently on each task and each featuring half the number of hidden neurons (Fig. 4(c) in the main body text) and half the number of parameters (Fig. 4(d) in the main body text) of the metaplastic network. This choice allows verifying in this situation whether a metaplastic network performs better than a network partitioned into two parts, with each partition trained on one task, independently from the other. As the MNIST dataset is much larger than the USPS one, we follow the training protocol introduces in [10] and [11], where 2,000 training examples are used for MNIST and 1,800 for USPS. For this reason, we focus on relatively small neural networks. The small network is a convolutional neural network with three layers of 4×4 kernels with increasing feature maps of 6-10-15 (4,056 parameters) while the neural network with twice more neurons (Fig. 4(c)) has feature maps 12-20-30 (14,832 parameters). We can choose the dimensions to be 10-15-20 to obtain a network with approximately twice more parameters (8,160) (Fig. 4(d)).

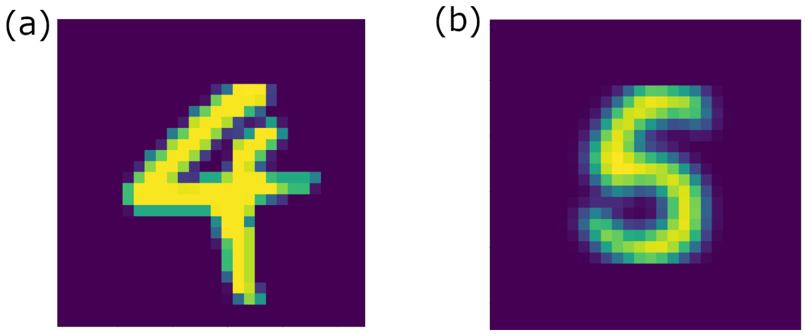

Supplementary Figure 7. (a) One MNIST training example, (b) One USPS training example.

Supplementary Note 11: Class Incremental Learning on CIFAR-10 and CIFAR-100 Features

In this note, we investigate a setting of class incremental learning, where a network learns different subsets of classes of the CIFAR-10 and CIFAR-100 datasets sequentially. We focus on the sequential training of the fully-connected layers of a convolutional neural network. This choice is motivated by the fact that the ability to extract features from visual input does not change across time presumably: for instance, one does not usually forget how to recognize shapes, but rather we can forget abstracted concepts.

To extract relevant features from the CIFAR-10 and CIFAR-100 datasets, we therefore use the convolutional layers of a ResNet-18 network [12], pretrained on the ImageNet dataset, and available in the PyTorch 1.1.0 library. This choice ensures that the feature extractor is fairly general, without having been trained on CIFAR images. We create a feature-extracted dataset of CIFAR-10 and CIFAR-100 by resizing CIFAR images from 32×32 to 220×220 pixels, and applying random crops of a 200×200 window, as well as random horizontal flips. We then perform ten passes through the training set of each dataset, resulting in 500,000 training images for each training sets. We perform only one pass through the test sets and do not apply data augmentation (we only resize the test images to 220×220 pixels and center-crop them to 200×200 pixels). The features obtained by this procedure are 512-dimensional vectors.

The architectures we use for learning the extracted features are binarized multilayer perceptrons of dimensions 512-2048-10 for CIFAR-10, and 512-2048-2048-100 for CIFAR-100. The results are shown in Supplementary Fig. 8 for datasets split into two subsets of classes. The subsets for CIFAR-10 are chosen by grouping together similar classes : subset 1 consists of vehicle classes and the horse class, while subset 2 consists of the remaining animal classes. For CIFAR-100, the subsets of classes are chosen randomly.

We consider three settings for CIFAR-10 and CIFAR-100. Figs. 8(a) and (d) show the training results for a non-metaplastic setting. We see that, when the network starts learning the second subset of classes, it forgets the first subset of classes rapidly and entirely.

The results for a metaplastic network with task dependent thresholds are shown in Figs. 8(b) and (e). The metaplasticity parameter m was optimized in each case by hyperparameter grid search. Learning in this situation is highly successful. For CIFAR-10, at the end of learning, accuracy on both subsets approaches the maximum accuracies at the end of subphases of Fig. 8(a). For CIFAR-100, accuracy on the first subset approaches the maximum one reached in Fig. 8(d). The accuracy on the second subset is also very high, but remains below the maximum one reached in Fig. 8(d). These results highlight the applicability of our metaplasticity approach to datasets more sophisticated than MNIST. However, this situation does not correspond to a truly incremental task learning situation, as the output is computed given information on the subset at hand.

For this reason, in Figs. 8(c) and (f), we use the technique of "instance normalization" [13] to avoid the task dependency through neurons thresholds. In this situation, during testing, it is not necessary for the network to know to which subset of classes the presented image belongs. The metaplasticity parameter m was again optimized in each case by hyperparameter grid search. We see that incremental learning is achieved, successfully, with final accuracies that do not, however, match the ones seen with task-dependent thresholds in Figs. 8(b) and (e), highlighting the difficulty of this training situation.

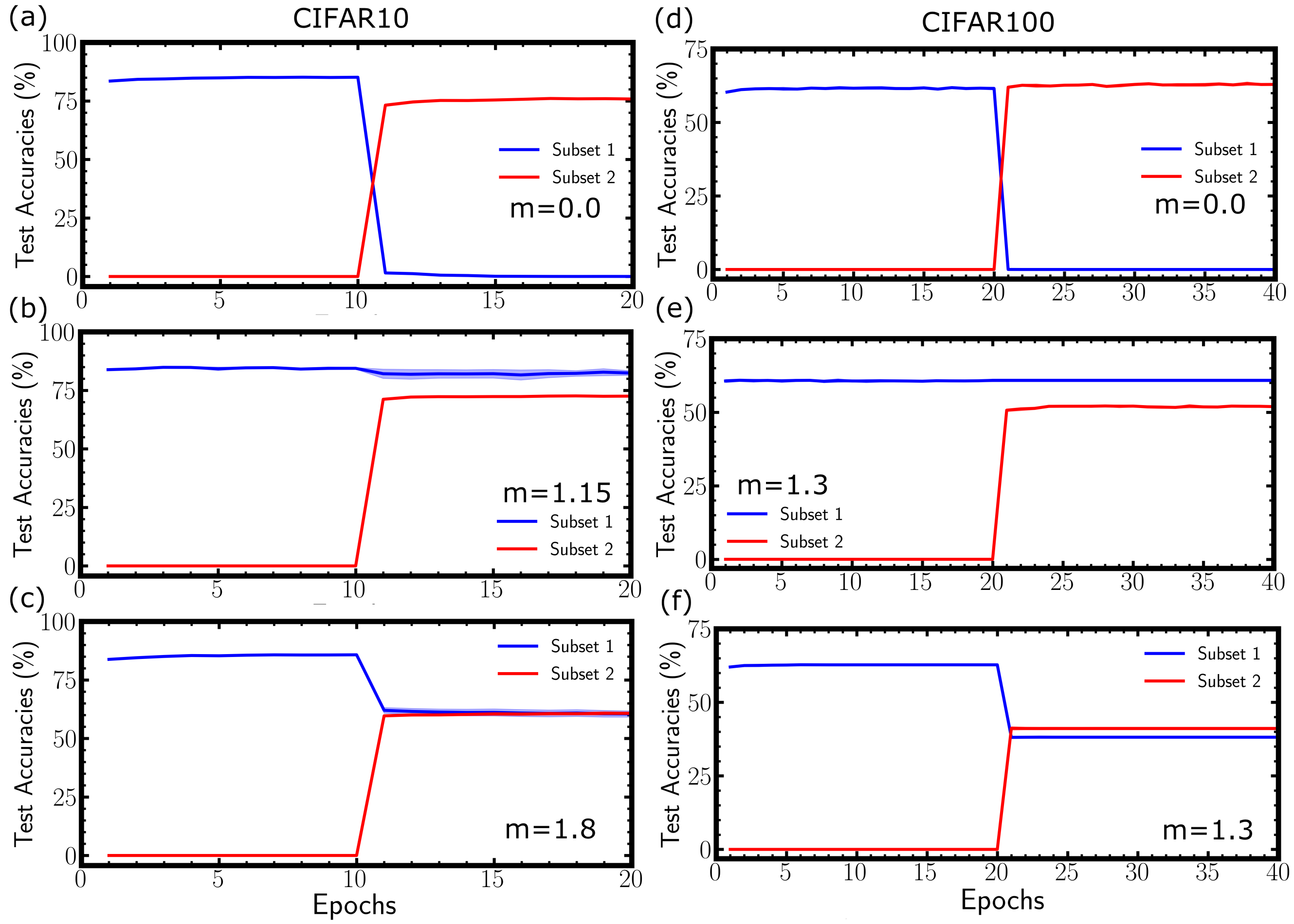

Supplementary Figure 8. Class Incremental Learning on CIFAR-10 features (a,b,c) and CIFAR-100 features (d,e,f) with the following settings : (a,d) Non-metaplastic (b,e) Metaplastic with task dependent neurons activation thresholds through Batch Normalization (c,f) No dependency on task (Instance Normalization). The curves are averaged over five runs and shadows stand for one standard deviation.

Supplementary References

1. Courbariaux, M., Hubara, I., Soudry, D., El-Yaniv, R. & Bengio, Y. Binarized neural networks: Training deep neural networks with weights and activations constrained to+ 1 or-1. *arXiv preprint arXiv:1602.02830* (2016).

2. Ioffe, S. & Szegedy, C. Batch normalization: Accelerating deep network training by reducing internal covariate shift. *arXiv preprint arXiv:1502.03167* (2015).

3. Kingma, D. P. & Ba, J. Adam: A method for stochastic optimization. *arXiv preprint arXiv:1412.6980* (2014).

4. Zenke, F., Poole, B. & Ganguli, S. Continual learning through synaptic intelligence. In *Proceedings of the 34th International Conference on Machine Learning-Volume 70*, 3987–3995 (JMLR. org, 2017).

5. Benna, M. K. & Fusi, S. Computational principles of synaptic memory consolidation. *Nat. neuroscience* **19**, 1697 (2016).

6. van de Ven, G. M. & Tolias, A. S. Three scenarios for continual learning. *arXiv preprint arXiv:1904.07734* (2019).

7. Fusi, S., Drew, P. J. & Abbott, L. F. Cascade models of synaptically stored memories. *Neuron* (2005).

8. Benna, M. K. & Fusi, S. Efficient online learning with low-precision synaptic variables. In *2017 51st Asilomar Conference on Signals, Systems, and Computers*, 1610–1614 (IEEE, 2017).

9. Xiao, H., Rasul, K. & Vollgraf, R. Fashion-mnist: a novel image dataset for benchmarking machine learning algorithms. *arXiv preprint arXiv:1708.07747* (2017).

10. Long, M., Wang, J., Ding, G., Sun, J. & Yu, P. S. Transfer feature learning with joint distribution adaptation. In *Proceedings of the IEEE international conference on computer vision*, 2200–2207 (2013).

11. Tzeng, E., Hoffman, J., Saenko, K. & Darrell, T. Adversarial discriminative domain adaptation. In *Proceedings of the IEEE conference on computer vision and pattern recognition*, 7167–7176 (2017).

12. He, K., Zhang, X., Ren, S. & Sun, J. Deep residual learning for image recognition. In *Proceedings of the IEEE conference on computer vision and pattern recognition*, 770–778 (2016).

13. Ulyanov, D., Vedaldi, A. & Lempitsky, V. Instance normalization: The missing ingredient for fast stylization. *arXiv preprint arXiv:1607.08022* (2016).
